# Supplementary material for: Binding Studies and Lead Generation of Pteridin-7(8H)-one Derivatives Targeting FLT3
Source: Int J Mol Sci. 2022 Jul 12;23(14):7696. doi: 10.3390/ijms23147696 (PMC9319409; doi:10.3390/ijms23147696)
Supplement: Supplementary file 1 [file ijms-23-07696-s001.zip › ijms-1797232-supplementary.pdf]

Supporting Information

Binding studies and lead generation of pteridin-7(8*H*)-one derivatives targeting FLT3

Suparna Ghosh[1], Seung Joo Cho[1,2,*]
[1]Department of Biomedical Sciences, College of Medicine, Chosun University, Gwangju 501-759, Korea; s.ghosh@chosun.kr
[2]Department of Cellular and Molecular Medicine, College of Medicine, Chosun University, Gwangju 501-759, Korea
*Correspondence: chosj@chosun.ac.kr; Tel.: +82-010-5479-1010 or +82-62-230-7482

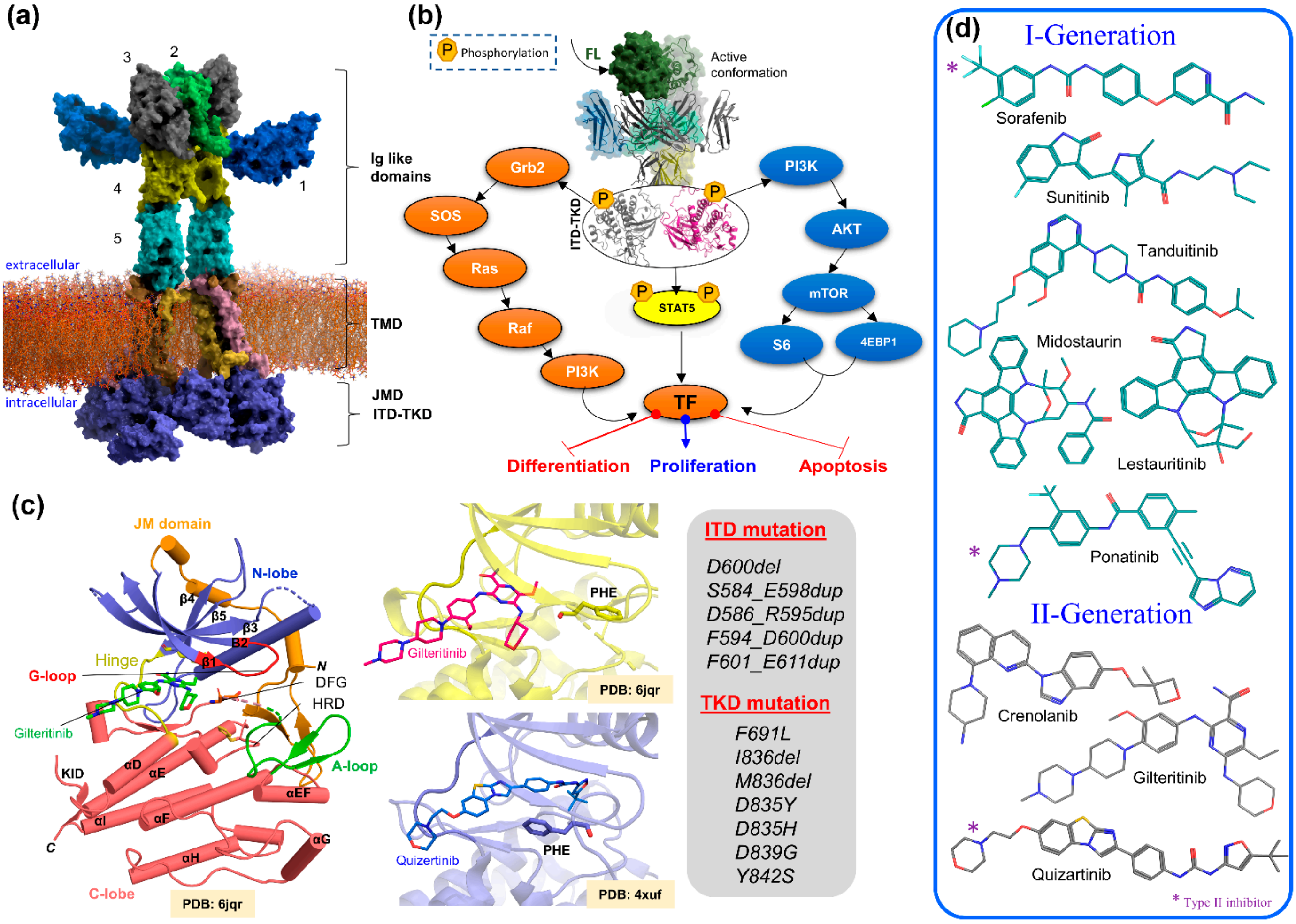

Figure S1. Structure, function, and inhibitory mechanisms of FLT3. (a) A model representation of the FLT3 receptor that is bound to the membrane, consists of five immunoglobulin-like domains (PDB ID 3QS9), a transmembrane domain (PDB ID 4I0U), a juxtamembrane domain, and two kinase domains (PDB ID 6JQR). (b) Down-signaling of FLT3 by Ras/Raf/MAPK, STAT5, and Akt/mTOR pathways upon binding to the FL ligand. (c) X-ray structure of the gilteritinib (type I) bound FLT3 kinase domain (PDB ID 6JQR). The binding modes of type I and type II (quizertinib) in the active (DFG-in) and inactive (DFG-out) configuration. The frequently found ITD and TKD mutations are listed in the inset box. (d) First generation and second-generation type I and type II (* sign) FLT3 inhibitors displayed in green and grey C-atoms.

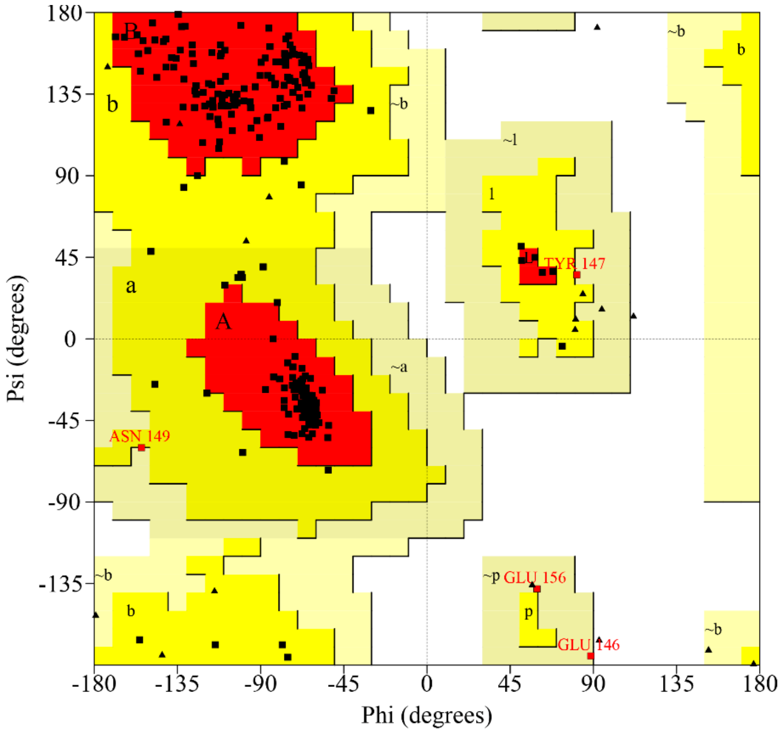

Figure S2. Ramachandran plot analysis of modeled FLT3. All residues fall within well-accepted regions.

Table S1. Molecular docking by Surflex dock and RMSD calculation of the crystallographic pose of gilteritinib using the LigRMSD web server.

Compounds	Total Score	Crash	Polar	similarity	RMSD (Å)
C01	6.32	-1.12	2.17	0.53	2.14
C03	6.61	-1.25	1.97	0.50	2.13
C06	7.42	-2.36	2.19	0.43	1.66
C17	8.41	-2.23	0.91	0.34	1.71
C22	6.13	-0.73	1.34	0.49	1.99
C28	7.19	-1.01	2.22	0.50	1.70
C31	6.93	-0.99	1.90	0.50	2.21

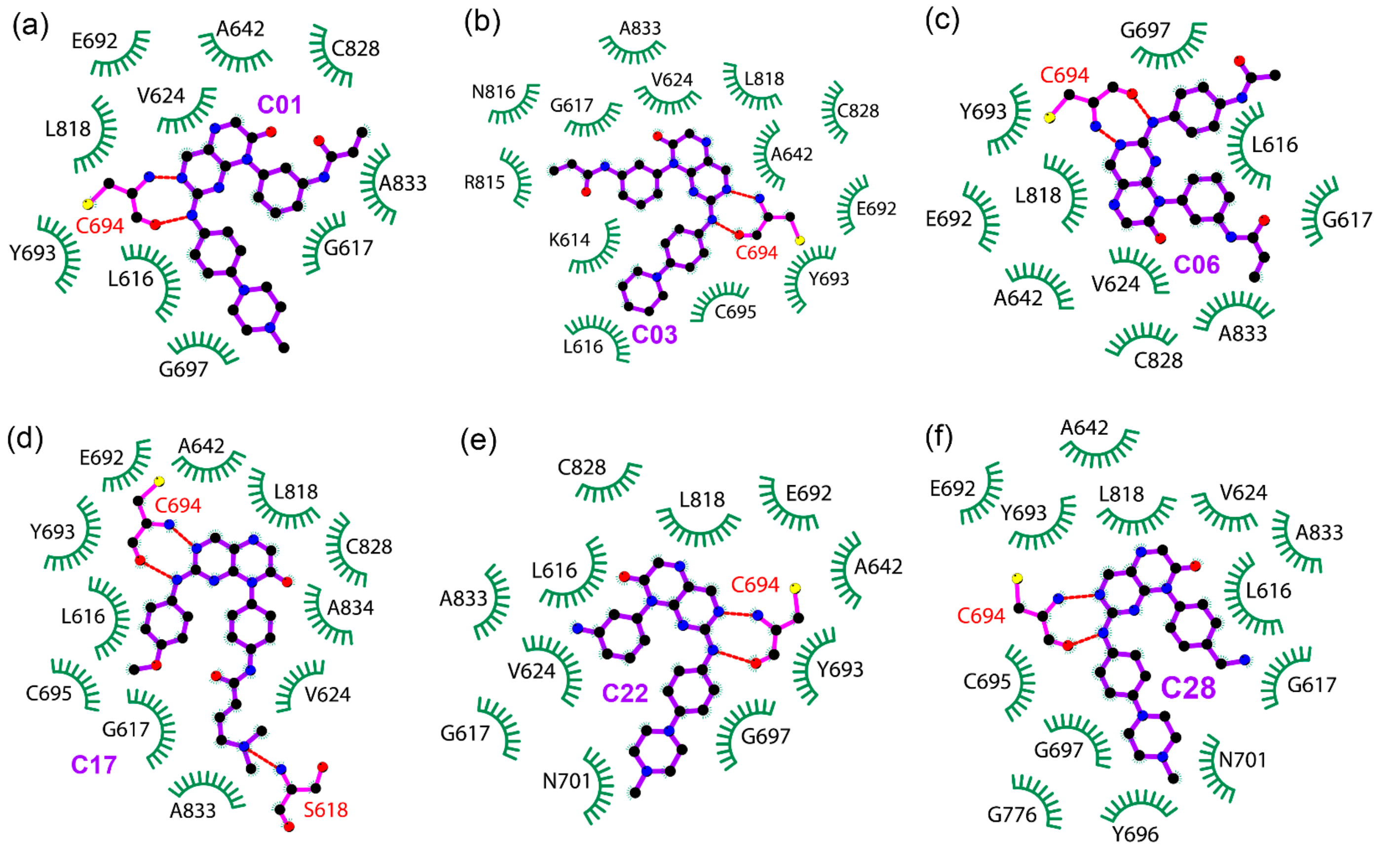

Figure S3. 2D docking interaction analysis of (a) FLT3-C01, (b) FLT3-C01, (c) FLT3-C06, (d) FLT3-C17, (e) FLT3-C22 and (f) FLT3-C28 complexes. The H-bond interactions are shown in red dotted lines. The hydrophobic interacting residues are shown in green.

Table S2. MM-PB/GBSA binding free energy terms in kcal/mol

Complexes	E_{vdW} (±SD)	E_{EL} (±SD)	E_{GB} (±SD)	E_{SURF} (±SD)	E_{gas} (±SD)	E_{solv} (±SD)	E_{TOTAL} (±SD)	TΔS	$\Delta G_{MM-PB/GBSA}$ (±SD)
FLT3-C01	-51.58 ± 2.72	-21.31 ± 5.81	43.74 ± 4.01	-6.07 ± 0.32	-72.89 ± 5.85	37.67 ± 3.96	-35.22 ± 4.03	12.51 ± 1.56	-22.70 ± 4.32
FLT3-C03	-52.45 ± 2.40	-29.53 ± 4.49	49.79 ± 3.98	-6.12 ± 0.24	-81.99 ± 4.92	43.67 ± 3.95	-38.31 ± 3.06	15.68 ± 0.04	-22.62 ± 3.06
FLT3-C06	-52.90 ± 2.54	-27.56 ± 6.44	48.97 ± 6.15	-6.31 ± 0.19	-80.47 ± 6.85	42.66 ± 6.10	-37.81 ± 3.25	16.09 ± 0.03	-21.71 ± 3.25
FLT3-C17	-52.03 ± 2.56	-20.74 ± 7.09	40.48 ± 5.92	-5.73 ± 0.23	-72.78 ± 6.64	34.74 ± 5.85	-38.04 ± 3.44	11.19 ± 2.06	-26.84 ± 4.01
FLT3-C22	-50.34 ± 3.07	-20.44 ± 3.99	39.62 ± 2.81	-5.83 ± 0.27	-70.78 ± 4.89	33.78 ± 2.81	-37.00 ± 3.68	6.17 ± 0.04	-30.83 ± 3.68
FLT3-C28	-53.98 ± 2.53	-29.84 ± 3.67	48.08 ± 3.43	-6.43 ± 0.24	-83.83 ± 4.71	41.65 ± 3.32	-42.18 ± 3.32	12.20 ± 1.22	-30.97 ± 3.46
FLT3-C31	-53.90 ± 2.46	-27.85 ± 4.63	45.00 ± 3.82	-6.05 ± 0.30	-81.76 ± 5.35	38.94 ± 3.69	-42.81 ± 3.13	10.65 ± 0.06	-32.15 ± 3.13
$FLT3_{D835Y}$-C31	-50.79 ± 2.35	-23.52 ± 4.09	40.73 ± 3.23	-5.70 ± 0.19	-74.31 ± 4.61	35.02 ± 3.18	-39.29 ± 2.60	8.74 ± 2.29	-30.54 ± 3.47

Table S3. Per-residue MM-PB/GBSA binding energy decomposition in kcal/mol

Residues	Complexes							
	**FLT3-C31 (±SD)**	**FLT3$_{D835Y}$-C31 (±SD)**	**FLT3-C01 (±SD)**	**FLT3-C03 (±SD)**	**FLT3-C06 (±SD)**	**FLT3-C17 (±SD)**	**FLT3-C22 (±SD)**	**FLT3-C28 (±SD)**
**K614**	-0.31 ±0.43	-0.26 ± 0.36	-0.40 ± 0.81	NA	NA	NA	NA	NA
**L616**	-3.20 ±0.48	-3.20 ± 0.46	-2.62 ± 0.56	-3.00± 0.51	-2.90 ± 0.48	-2.96 ± 0.47	-3.49 ± 0.46	-2.88 ± 0.54
**G617**	NA	-0.27 ± 0.14	- 0.40 ± 0.13	-0.48 ±0.17	-0.26 ± 0.11	-0.63 ± 0.21	NA	-0.57 ± 0.17
**V624**	-1.83 ±0.26	-1.16 ± 0.30	-1.68 ± 0.24	-1.80 ±0.31	-1.53 ± 0.29	-1.44 ± 0.28	-1.77 ± 0.27	-1.95 ± 0.27
**A642**	-0.98 ±0.26	-1.00 ± 0.25	-0.93 ± 0.18	-0.89 ±0.16	-1.02 ± 0.23	-1.01 ± 0.17	-1.10 ± 0.26	-1.08 ± 0.19
**E692**	-0.28 ±0.30	-0.26 ± 0.32	-0.25 ± 0.21	NA	-0.02 ± 0.40	-0.11 ± 0.34	NA	-0.16 ± 0.35
**Y693**	-1.88 ±0.39	-1.92 ± 0.34	-1.57 ± 0.35	-1.72 ±0.35	-1.74 ± 0.41	-1.99 ± 0.35	-1.94 ± 0.33	-1.78 ± 0.36
**C694**	-2.50 ±0.77	-2.81 ± 0.66	-3.00 ± 0.60	-2.94 ±0.59	-2.76 ± 0.61	-3.21 ± 0.56	-2.82 ± 0.59	-2.77 ± 0.64
**C695**	-0.37 ±0.15	-0.50 ± 0.15	NA	NA	NA	NA	NA	-0.34 ± 0.18
**Y696**	NA	NA	NA	NA	NA	NA	NA	-1.02 ± 0.25
**G697**	-2.25 ±0.29	2.09 ± 0.29	-1.66 ± 0.28	-1.55 ±0.27	-1.54 ± 0.23	-0.32 ± 0.13	-1.79 ± 0.29	-2.27 ± 0.32
**N701**	NA	NA	NA	NA	NA	NA	NA	-1.02 ± 0.25
**L818**	-2.63 ±0.34	-2.50 ± 0.30	-2.39 ± 0.25	-2.41 ±0.28	-2.80 ± 0.28	-1.65 ± 0.24	-2.46 ± 0.32	-2.94 ± 0.33
**C828**	NA	-1.35 ± 0.21	-1.02 ± 0.17	0.96 ±0.21	-1.10 ± 0.33	-2.16 ± 0.32	-1.25 ± 0.21	-1.06 ± 0.16
**D829**	NA	NA	-0.06 ± 0.23	NA	NA	-1.15 ± 0.21	NA	NA
**F830**	-1.58 ±0.34	-0.95 ± 0.40	-2.56 ± 0.50	-2.61 ±0.47	-2.57 ± 0.44	-1.63 ± 0.33	-1.28 ± 0.28	-1.22 ± 0.39
**A833**	NA	NA	-0.16 ± 0.05	NA	-0.11 ± 0.04	-0.94 ± 0.23	NA	-1.09 ± 0.32

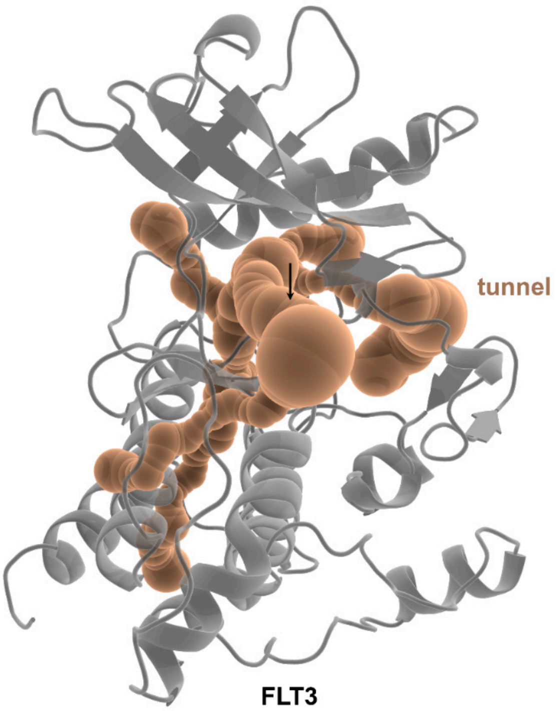

Figure S4. Caver analysis to determine the unbinding tunnel (arrow sign) from the binding pocket

Table S4. Rupture force (F_{MAX}) and LIE terms from FPL simulations. Except for the rupture force (F_{MAX}), all binding energy terms are in kcal/mol

Complexes	F_{MAX} (pN)	Ligands in Solvent		Ligands in Complex		ΔG_{LIE} (±SD)
		$\frac{1}{2}\Delta E_{coul}$ (±SD)	$\frac{1}{2}\Delta E_{vdW}$ (±SD)	$\frac{1}{2}\Delta E_{coul}$ (±SD)	$\frac{1}{2}\Delta E_{vdW}$ (±SD)	
FLT3-C01	221.40	-34.30 ± 2.55	-16.76 ± 2.69	-14.12 ± 2.86	-6.18 ±1.10	-28.76 ± 4.80
FLT3-C03	441.13	-27.38 ± 2.55	-17.22 ± 2.48	-11.52 ± 1.75	-5.90 ±1.10	-27.18 ± 4.11
FLT3-C06	391.61	-35.32 ± 2.19	-15.64 ± 3.10	-15.76 ± 1.86	-6.49 ± 1.86	-28.71 ± 4.61
FLT3-C17	428.75	-32.12 ±1.85	-18.19 ± 1.44	-14.31 ± 2.79	-5.03 ± 1.86	-30.65 ± 4.09
FLT3-C22	475.17	-28.23 ± 1.31	-15.68 ± 2.38	-10.61 ± 3.62	-4.65 ± 2.48	-30.97 ± 3.23
FLT3-C28	441.13	-29.57 ± 1.10	-16.84 ± 1.44	-12.36 ± 2.58	-3.70 ± 0.16	-30.35 ± 4.51
FLT3-C31	537.07	-26.79 ± 1.90	-17.65 ± 2.51	-11.79 ± 1.96	-5.48 ± 2.58	-28.92 ± 4.51
$FLT3_{D835Y}$-C31	453.51	-26.28 ± 2.38	-17.80 ± 1.34	-10.82 ± 1.90	-5.34 ± 2.33	-28.17 ± 4.06

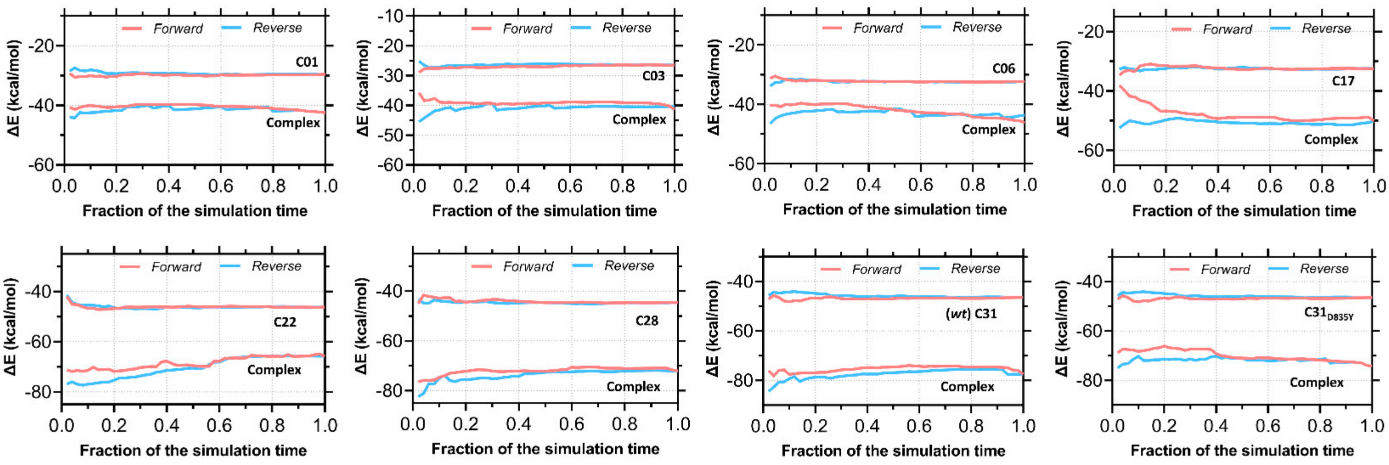

Figure S5. Energy convergence plots from the FEP simulation over the fraction of simulation time

Table S5. FEP binding energy terms in kcal/mol

Complexes	Ligands in Solvent			Ligands in Complex			ΔG_{FEP} (±SD)
	ΔE_{coul} (±SD)	ΔE_{vdW} (±SD)	ΔE_{Total} (±SD)	ΔE_{coul} (±SD)	ΔE_{vdW} (±SD)	ΔE_{Total} (±SD)	
FLT3-C01	-26.47 ±0.09	-1.34 ±0.38	-27.82 ±0.39	-14.49 ±0.52	-28.16 ±0.09	-42.65 ±0.53	-14.83 ±0.65
FLT3-C03	-25.45 ±0.08	-1.15 ±0.33	-26.60 ±0.34	-16.94 ±0.43	-24.30 ±0.08	-41.24 ±0.43	-14.64 ±0.54
FLT3-C06	-31.37 ±0.09	-1.19 ±0.28	-32.57 ±0.30	-17.43 ±0.28	-28.82 ±0.14	-46.25 ±0.32	-13.68 ±0.43
FLT3-C17	-32.68 ±0.16	0.35 ±0.28	-33.03 ±0.33	-20.98 ±0.22	-28.82 ±0.03	-49.80 ±0.23	-16.77 ±0.40
FLT3-C22	-24.83 ±0.05	-1.39 ±0.32	-26.22 ±0.33	-20.89 ±0.11	-20.37 ±0.31	-41.26 ±0.33	-15.04 ±0.46
FLT3-C28	-24.60 ±0.06	-1.42 ±0.38	-26.02 ±0.38	-24.03 ±0.35	-21.59 ±0.11	-45.63 ±0.37	-17.61 ±0.53
FLT3-C31	-26.17 ±0.06	-1.72 ±0.30	-27.90 ±0.30	-21.42 ±0.09	-24.35 ±0.30	-45.77 ±0.31	-17.87 ±0.43
$FLT3_{D835Y}$-C31	-26.17 ±0.06	-1.72 ±0.30	-27.90 ±0.30	-24.10 ±0.11	-20.61 ±0.21	-44.72 ±0.24	-16.82 ±0.38

Table S6. Dataset compounds and their inhibitory activities. The core common chemical structure is shown as 'Structure A'. The chemical substitutions are shown in R1, R2 and R3 columns. The test set compounds are shown in the (*) sign

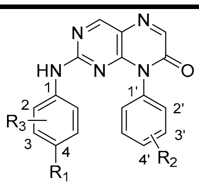

Structure A

#Cpds	R_1	R_2	R_3	pIC_{50}	#Cpds	R_1	R_2	R_3	pIC_{50}
01*	4-methylpiperazin-1-yl	3'–NH–C(O)–CH=CH$_2$	H	6.50	19	OMe	4'–NH_2	H	7.53
02	pyrrolidin-1-yl	3'–NH–C(O)–CH=CH$_2$	H	5.81	20	4-methylpiperazin-1-yl	3'–NH–C(O)–CH=CH$_2$	2-OMe	5.88
03*	piperidin-1-yl	3'–NH–C(O)–CH=CH$_2$	H	5.97	21	4-methylpiperazin-1-yl	4'–NH–C(O)–CH=CH$_2$	2-OMe	6.81
04	N(CH$_2$CH$_3$)$_2$	3'–NH–C(O)–CH=CH$_2$	H	5.75	22	4-methylpiperazin-1-yl	3'–NH_2	H	8.21
05	CH$_2$–C(O)–NH_2	3'–NH–C(O)–CH=CH$_2$	H	5.54	23*	4-methylpiperazin-1-yl	4'–NH_2	H	8.05
06	NH–C(O)–CH_3	3'–NH–C(O)–CH=CH$_2$	H	5.26	24	OMe	4'–NH–C(O)–CH_3	H	6.89
07*	OMe	3'–NH–C(O)–CH=CH$_2$	H	5.72	25	OMe	4'–NH–S(O$_2$)–CH_3	H	6.59
08	H	3'–NH–C(O)–CH=CH$_2$	H	5.53	26	4-methylpiperazin-1-yl	4'–NH–C(O)–CH_3	H	7.65
09	Cl	3'–NH–C(O)–CH=CH$_2$	H	5.44	27*	4-methylpiperazin-1-yl	4'–NH–S(O$_2$)–CH_3	H	7.49
10	morpholin-4-yl	3'–NH–C(O)–CH=CH$_2$	H	5.94	28	4-methylpiperazin-1-yl	4'–CH_2NH_2	H	7.42
11*	4-methylpiperazin-1-yl	4'–NH–C(O)–CH=CH$_2$	H	7.32	29	4-methylpiperazin-1-yl	4'–N(piperazine, NH)	H	8.08
12	OMe	4'–NH–C(O)–CH=CH$_2$	H	6.96	30	OMe	4'–NH_2	2–Me	6.45
13*	H	4'–NH–C(O)–CH=CH$_2$	H	6.78	31	4-methylpiperazin-1-yl	4'–NH_2	3–Me	8.80
14	Cl	4'–NH–C(O)–CH=CH$_2$	H	6.41	31$_{(D835Y)}$	”	”	”	8.69
15	morpholin-4-yl	4'–NH–C(O)–CH=CH$_2$	H	6.76	32*	4-methylpiperazin-1-yl	4'–NH_2	3–OMe	8.49
16*	OMe	3'–NH–C(O)–CH=CH–CH$_2$–N(CH$_3$)$_2$	H	5.35	33	4-methylpiperazin-1-yl	4'–NH_2	3–Cl	8.28
17	OMe	4'–NH–C(O)–CH=CH–CH$_2$–N(CH$_3$)$_2$	H	6.73	34	4-methylpiperazin-1-yl	4'–NH_2	3–Cl	7.55
18	OMe	3'–NH_2	H	7.46	35	4-methylpiperazin-1-yl	4'–NH_2	3–F	7.44

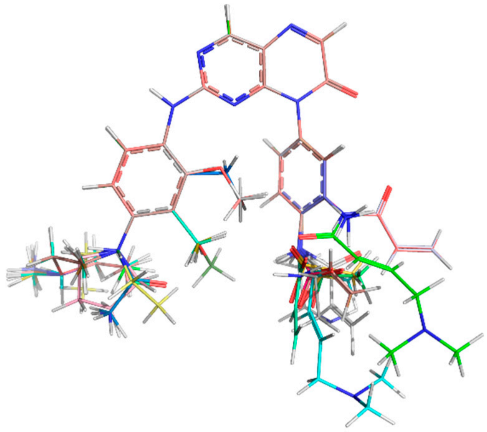

Figure S6: Molecular alignment of the dataset compounds over the template molecule C31

Table S7: Statistical analysis to generate the best CoMSIA model using various combinations of the descriptors field

CoMSIA	q^2	ONC	SEP	r^2	SEE	F-value	Field Contribution				
							S	E	H	A	D
S	0.840	3	0.434	0.924	0.299	89.24	100	-	-	-	-
E	0.704	2	0.578	0.871	0.381	77.93	-	100	-	-	-
H	0.749	2	0.532	0.843	0.421	61.77	-	-	100	-	-
A	0.205	5	1.016	0.523	0.786	4.38	-	-	-	100	-
D	0.597	5	0.729	0.914	0.334	42.56	-	-	-	-	100
SE	0.799	6	0.524	0.979	0.171	144.67	44.6	55.4	-	-	-
EH	0.751	3	0.542	0.920	0.307	84.19	-	51.4	48.6	-	-
EA	0.755	5	0.564	0.945	0.267	68.88	-	81.0	-	19.0	-
ED	0.740	2	0.542	0.889	0.353	92.42	-	50.0	-	-	50.0
SH	0.844	4	0.439	0.961	0.219	129.98	48.8	-	53.2	-	-
SA	0.816	6	0.501	0.844	0.429	39.71	72.2	-	-	27.8	-
SD	0.777	2	0.502	0.891	0.351	93.80	41.8	-	-	-	58.2
HA	0.783	5	0.531	0.908	0.345	39.61	-	-	75.4	24.6	-
HD	0.781	2	0.497	0.903	0.331	106.62	-	-	49.9	-	50.1
AD	0.623	5	0.699	0.931	0.299	53.94	-	-	-	35.1	64.9
SHE	0.806	3	0.478	0.941	0.263	117.98	27.9	36.6	35.5	-	-
SEA	0.801	5	0.508	0.949	0.257	74.74	35.9	52.7	-	11.4	-
SED	0.795	6	0.530	0.981	0.163	159.43	27.2	39.9	-	-	32.8
EHA	0.787	4	0.512	0.934	0.285	74.52	-	43.0	41.8	15.2	-
EHD	0.782	2	0.496	0.900	0.336	103.62	-	33.5	31.7	-	34.8

SHA	0.835	5	0.462	0.953	0.248	80.36	36.4	-	48.8	14.8	-
SHD	0.802	2	0.467	0.901	0.334	104.80	27.7	-	33.6	-	38.7
EAD	0.764	6	0.568	0.973	0.193	113.22	-	49.4	-	13.9	36.7
HAD	0.771	4	0.532	0.940	0.240	83.43	-	-	43.6	17.0	39.4
SEHD	0.800	3	0.485	0.939	0.269	112.15	19.3	24.6	24.4	-	31.7
SEHA	0.814	5	0.491	0.956	0.238	87.49	24.8	31.8	31.7	11.7	-
SEAD	0.795	6	0.529	0.980	0.166	153.23	21.5	38.7	-	9.3	30.5
EHAD	0.802	5	0.507	0.969	0.200	125.21	-	31.9	29.9	12.5	25.7
SHAD	0.806	3	0.478	0.936	0.276	106.51	23.1	-	28.8	14.5	33.7
SEHAD	0.809	3	0.475	0.945	0.255	125.52	17.3	21.1	21.5	13.4	26.7

q^2: squared cross-validated correlation coefficient; **ONC**: optimal number of components; **SEP**: standard error of prediction; ***r^2***: squared correlation coefficient; **SEE**: standard error of estimation; **F-value**: F-test value; ***r^2_{pred}***: predictive r^2; **S**: Steric; **E**: Electrostatic; **H**: Hydrophobic; **A**: H-bond acceptor; **D**: H-bond donor. Green highlighted box showing the final selection of the CoMSIA model.

Table S8. Actual pIC_{50} vs. predicted pIC_{50} values with their residuals of selected CoMFA and CoMSIA (SH) models. Green highlighted box showing the Test set compounds

Cpd. No.	CoMFA			CoMSIA(SH)		
	Actual pIC_{50}	Predicted pIC_{50}	Residual	Actual pIC_{50}	Predicted pIC_{50}	Residual
C06	5.2637	5.63	-0.3663	5.2637	5.698	-0.4343
C09	5.445	5.521	-0.076	5.445	5.503	-0.058
C08	5.5346	5.643	-0.1084	5.5346	5.575	-0.0404
C05	5.5439	5.423	0.1209	5.5439	5.578	-0.0341
C04	5.7577	5.836	-0.0783	5.7577	5.834	-0.0763
C02	5.8159	5.819	-0.0031	5.8159	5.734	0.0819
C20	5.8804	6.058	-0.1776	5.8804	6.014	-0.1336
C10	5.9446	5.758	0.1866	5.9446	5.95	-0.0054
C14	6.4157	6.641	-0.2253	6.4157	6.676	-0.2603
C30	6.4597	7.491	-1.0313	6.4597	7.5	-1.0403
C25	6.5935	6.498	0.0955	6.5935	6.678	-0.0845
C17	6.7352	6.803	-0.0678	6.7352	6.69	0.0452
C15	6.762	6.865	-0.103	6.762	7.137	-0.375
C21	6.8153	6.815	0.0003	6.8153	6.777	0.0383
C24	6.8962	6.89	0.0062	6.8962	6.843	0.0532
C12	6.9666	6.822	0.1446	6.9666	6.939	0.0276

C28	7.4295	7.516	-0.0865	7.4295	7.515	-0.0855
C35	7.4461	7.808	-0.3619	7.4461	8.136	-0.6899
C18	7.4685	6.895	0.5735	7.4685	6.808	0.6605
C19	7.5376	7.553	-0.0154	7.5376	7.412	0.1256
C34	7.5575	7.522	0.0355	7.5575	7.517	0.0405
C26	7.6536	8.033	-0.3794	7.6536	7.778	-0.1244
C29	8.0862	8.201	-0.1148	8.0862	7.981	0.1052
C22	8.2125	7.452	0.7605	8.2125	7.423	0.7895
C33	8.2807	7.981	0.2997	8.2807	8.177	0.1037
C31	8.8069	8.733	0.0739	8.8069	8.474	0.3329
C16	5.3553	5.328	0.0273	5.3553	5.289	0.0663
C07	5.7284	5.791	-0.0626	5.7284	5.758	-0.0296
C03	5.9763	6.007	-0.0307	5.9763	5.874	0.1023
C01	6.5058	6.273	0.2328	6.5058	6.274	0.2318
C13	6.7825	6.757	0.0255	6.7825	6.748	0.0345
C11	7.3279	8.142	-0.8141	7.3279	8.075	-0.7471
C27	7.4949	7.582	-0.0871	7.4949	7.15	0.3449
C23	8.0585	7.971	0.0875	8.0585	8.081	-0.0225
C32	8.4962	8.43	0.0662	8.4962	8.472	0.0242

Table S9. SAR and MD assisted designed compounds in SMILES format

SMILES
NC1=CC=C(C=C1)N2C(C=NC3=C2N=C(NC4=CC(CC)=C(N5CCN(C)CC5)C=C4)N=C3)=O D01
NC1=CC=C(C=C1)N2C(C=NC3=C2N=C(NC4=CC(C(C)C)=C(N5CCN(C)CC5)C=C4)N=C3)=O D02
CC1=CC=C(C=C1)N2C(C=NC3=C2N=C(NC4=CC(C(C)C)=C(N5CCN(C)CC5)C=C4)N=C3)=O D03
O=C1C=NC2=C(N=C(NC3=CC(C(C)C)=C(N4CCN(C)CC4)C=C3)N=C2)N1C5=CC=CC=C5 D04
CC(C)C1=C(N2CCN(C3CC3)CC2)C=CC(NC(N=C4)=NC5=C4N=CC(N5C6=CC=CC(C)=C6)=O)=C1 D05
CC(C)C1=C(N2CCN(CC3CC3)CC2)C=CC(NC(N=C4)=NC5=C4N=CC(N5C6=CC=CC(C)=C6)=O)=C1 D06
CC(C)C1=C(N2CCN(CC3CC3)CC2)C=CC(NC(N=C4)=NC5=C4N=CC(N5C6=CC=CC=C6)=O)=C1 D07
CC(C)C1=C(N2CCN(C3CC3)CC2)C=CC(NC(N=C4)=NC5=C4N=CC(N5C6=CC=CC=C6)=O)=C1 D08
O=C1C=NC2=C(N=C(NC3=CC=C(N4CCN(C5CC5)CC4)C=C3)N=C2)N1C6=CC=CC(C)=C6 D09
O=C1C=NC2=C(N=C(NC3=CC=C(N4CCN(C5CC5)CC4)C=C3)N=C2)N1C6=CC=CC(F)=C6 D10
O=C1C=NC2=C(N=C(NC3=CC=C(N4CCN(C5CC5)CC4)C=C3)N=C2)N1C6=CC=CC(Cl)=C6 D11
O=C1C=NC2=C(N=C(NC3=CC=C(N4CCN(C5CC5)CC4)C=C3)N=C2)N1C6=CC=CC(Br)=C6 D12
O=C1C=NC2=C(N=C(NC3=CC(F)=C(N4CCN(C5CC5)CC4)C=C3)N=C2)N1C6=CC=CC=C6 D13
O=C1C=NC2=C(N=C(NC3=CC(Cl)=C(N4CCN(C5CC5)CC4)C=C3)N=C2)N1C6=CC=CC=C6 D14
O=C1C=NC2=C(N=C(NC3=CC(C(C)C)=C(N4CCN(C)CC4)C=C3)N=C2)N1C5=CC=CC(C)=C5 D15
O=C1C=NC2=C(N=C(NC3=CC(C(C)C)=C(N4CCN(C)CC4)C=C3)N=C2)N1C5=CC=CC=C5C D16
NC1C=NC2=C(N=C(NC3=CC(C(C)C)=C(N4CCN(C)CC4)C=C3)N=C2)N1C5=CC=CC=C5C D17
CN(CC1)CCN1C(C=C2)=C(C(C)C)C=C2NC(N=C3)=NC4=C3N=CCN4C5=CC(C=CC5)=O D18
O=C1C=NC2=C(N=C(NC3=CC(Br)=C(N4CCN(C5CC5)CC4)C=C3)N=C2)N1C6=CC=CC=C6 D19
O=C1C=NC2=C(N=C(NC3=CC(CCl)=C(N4CCN(C5CC5)CC4)C=C3)N=C2)N1C6=CC=CC=C6 D20

O=C1C=NC2=C(N=C(NC3=CC(CCl)=C(N4CCN(C5CC5)CC4)C=C3)N=C2)N1C6=CC=C(N)C=C6	D21
O=C1C=NC2=C(N=C(NC3=CC(CF)=C(N4CCN(C5CC5)CC4)C=C3)N=C2)N1C6=CC=C(N)C=C6	D22
O=C1C=NC2=C(N=C(NC3=CC(CF)=C(N4CCN(C5CC5)CC4)C=C3)N=C2)N1C6=CC=CC(N)=C6	D23
O=C1C=NC2=C(N=C(NC3=CC=C(N4CCN(CC5CC5)CC4)C=C3)N=C2)N1C6=CC=CC(F)=C6	D24
CN(CC1)CCN1C(C=C2)=C(C(C)C)C=C2NC(N=C3)=NC4=C3N=CC(N)N4C5=CC(C=CC5)=O	D25
CN(CC1)CCN1C(C=C2)=C(C(C)C)C=C2NC(N=C3)=NC4=C3N=CC(N)N4C5=CC=CC[C@H]5C(F)(F)F	D26
CC(C)C1=C(N2CCN(CC)CC2)C=CC(NC(N=C3)=NC4=C3N=CC(N)N4C5=CC=CC[C@H]5C(F)(F)F)=C1	D27
O=C1C=NC2=C(N=C(NC3=CC=C(N4CCN(CC5CC5)CC4)C=C3)N=C2)N1C6=CC=CC(Cl)=C6	D28
O=C1C=NC2=C(N=C(NC3=CC=C(N4CCN(CC5CC5)CC4)C=C3)N=C2)N1C6=CC=CC(Br)=C6	D29
O=C1C=NC2=C(N=C(NC3=CC(F)=C(N4CCN(CC5CC5)CC4)C=C3)N=C2)N1C6=CC=CC=C6	D30
O=C1C=NC2=C(N=C(NC3=CC(Cl)=C(N4CCN(CC5CC5)CC4)C=C3)N=C2)N1C6=CC=CC=C6	D31
O=C1C=NC2=C(N=C(NC3=CC(Br)=C(N4CCN(CC5CC5)CC4)C=C3)N=C2)N1C6=CC=CC=C6	D32
O=C1C=NC2=C(N=C(NC3=CC(CCl)=C(N4CCN(CC5CC5)CC4)C=C3)N=C2)N1C6=CC=CC=C6	D33
O=C1C=NC2=C(N=C(NC3=CC(CCl)=C(N4CCN(C5CC5)CC4)C=C3)N=C2)N1C6=CC=C(N)C=C6	D34
O=C1C=NC2=C(N=C(NC3=CC(CF)=C(N4CCN(CC5CC5)CC4)C=C3)N=C2)N1C6=CC=C(N)C=C6	D35
O=C1C=NC2=C(N=C(NC3=CC(CF)=C(N4CCN(CC5CC5)CC4)C=C3)N=C2)N1C6=CC=CC(N)=C6	D36
O=C1C=NC2=C(N=C(NC3=CC=C(N4CCN(CC5CC5)CC4)C=C3)N=C2)N1C6=CC(F)C=C6	D37
O=C1C=NC2=C(N=C(NC3=CC=C(N4CCN(CC5CC5)CC4)C=C3)N=C2)N1C6=CC(Cl)C=C6	D38
O=C1C=NC2=C(N=C(NC3=CC=C(N4CCN(CC5CC5)CC4)C=C3)N=C2)N1C6=CC(Br)C=C6	D39
O=C1C=NC2=C(N=C(NC3=CC(Cl)=C(N4CCN(CC5CC5)CC4)C=C3)N=C2)N1C6=CCC=C6	D40
O=C1C=NC2=C(N=C(NC3=CC(F)=C(N4CCN(CC5CC5)CC4)C=C3)N=C2)N1C6=CCC=C6	D41
O=C1C=NC2=C(N=C(NC3=CC(Br)=C(N4CCN(CC5CC5)CC4)C=C3)N=C2)N1C6=CCC=C6	D42
O=C1C=NC2=C(N=C(NC3=CC(CCl)=C(N4CCN(CC5CC5)CC4)C=C3)N=C2)N1C6=CCC=C6	D43
O=C1C=NC2=C(N=C(NC3=CC(CF)=C(N4CCN(CC5CC5)CC4)C=C3)N=C2)N1C6=CC(N)C=C6	D44
CC(C)C1=C(N2CCN(C(F)(F)F)CC2)C=CC(NC(N=C3)=NC4=C3N=CC(N4C5=CC=CC=C5)=O)=C1	D45
CC(C)C1=C(N2CCN(C(Cl)(Cl)Cl)CC2)C=CC(NC(N=C3)=NC4=C3N=CC(N4C5=CC=CCC5C)=O)=C1	D46
CC(C)C1=C(N2CCN(C(Cl)(Cl)Cl)CC2)C=CC(NC(N=C3)=NC4=C3N=CC(N4C5=CC=CC(C)=C5)=O)=C1	D47
O=C1C=NC2=C(N=C(NC3=CC(CBr)=C(N4CCN(CC5CC5)CC4)C=C3)N=C2)N1C6=CC(N)C=C6	D48
O=C1C=NC2=C(N=C(NC3=CC(C(C)(C)C)=C(N4CCN(CC5CC5)CC4)C=C3)N=C2)N1C6=CC(N)C=C6	D49
CC(C)C1=C(N2CCN(CC(F)(F)F)CC2)C=CC(NC(N=C3)=NC4=C3N=CC(N)N4C5=CC=CCC5C)=C1	D50

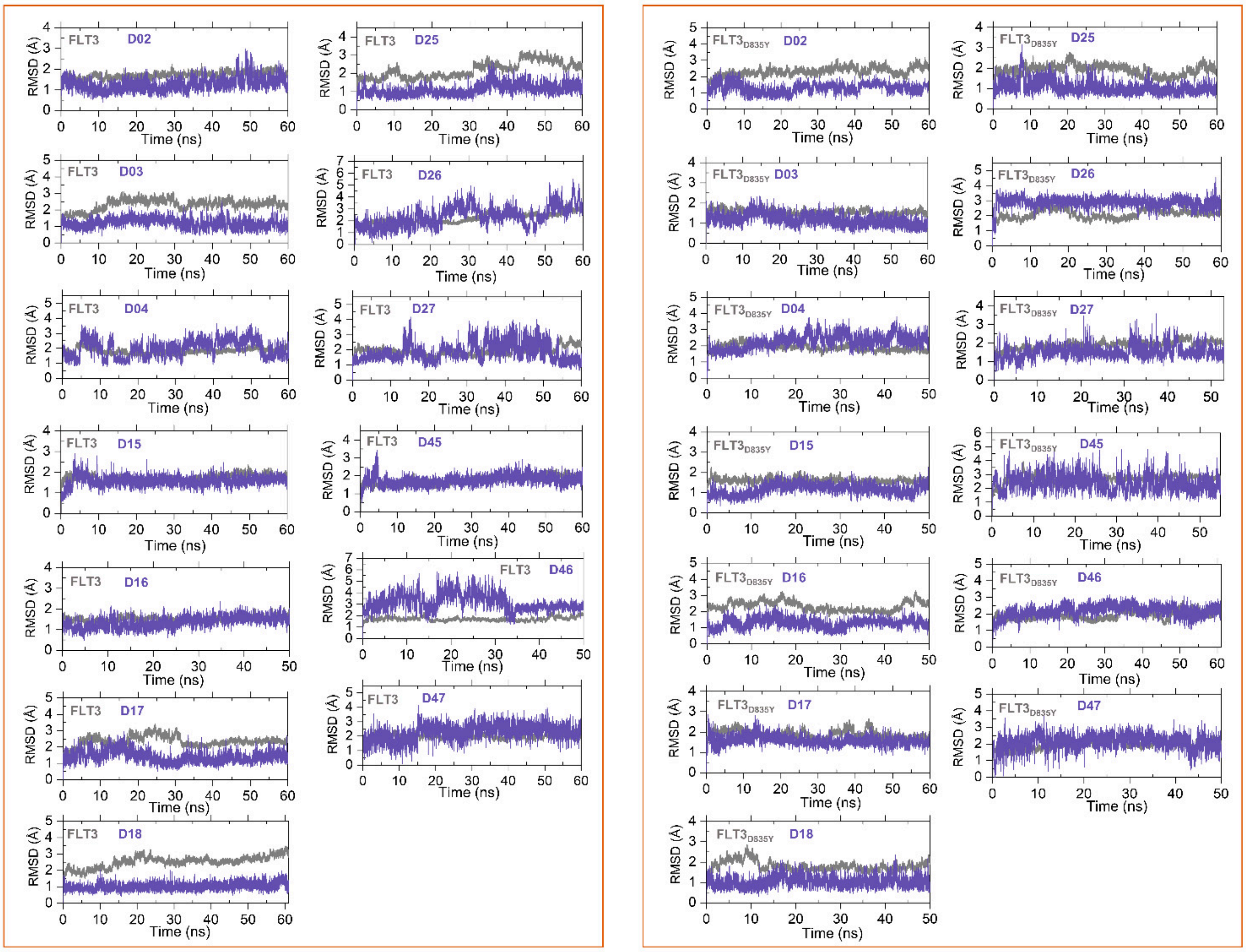

Figure S7: RMSD plots of the designed compounds in complex with wild-type and mutant FLT3

Table S10: MM-PB/GBSA binding energy terms of the designed compounds in complex with wild-type FLT3 in kcal/mol

Compounds	E_{vdW} (±SD)	E_{EL} (±SD)	E_{GB} (±SD)	E_{SURF} (±SD)	E_{gas} (±SD)	E_{solv} (±SD)	E_{TOTAL} (±SD)	TΔS	$\Delta G_{MM-PB/GBSA}$ (±SD)
D02	-52.08 ±2.41	-20.57 ±6.16	41.95 ±4.72	-5.77 ±0.26	-72.66 ±6.18	36.18 ±4.68	-36.48 ±3.58	8.26 ±0.05	-28.22 ±3.58
D03	-53.72 ±2.37	-22.58 ±3.02	41.93 ±1.81	-5.83 ±0.20	-76.29 ±3.46	36.09 ±1.76	-40.20 ±2.60	6.37 ±0.07	-33.82 ±2.60
D04	-48.71 ±2.35	-18.28 ±2.72	36.51 ±2.33	-5.13 ±0.16	-66.99 ±3.64	31.37 ±2.30	-35.62 ±2.40	4.82 ±0.40	-30.80 ±2.40
D15	-57.08 ±3.46	-23.53 ±3.13	43.12 ±3.10	-6.27 ±0.48	-80.61 ±4.69	36.85 ±2.82	-43.75 ±3.23	5.45 ±0.89	-38.30 ±3.35
D16	-54.58 ±2.41	-22.32 ±3.66	43.99 ±2.96	-5.75 ±0.17	-76.90 ±4.02	38.24 ±2.97	-38.66 ±2.79	7.86 ±0.04	-30.79 ±2.79
D17	-49.69 ±2.10	-9.95 ±2.65	28.29 ±2.10	-5.35 ±0.15	-59.65 ±3.87	22.93 ±2.02	-36.71 ±2.03	3.50 ±0.04	-33.21 ±2.03
D18	-51.04 ±2.19	-7.35 ±3.01	22.25 ±2.35	-5.36 ±0.17	-58.40 ±3.40	16.88 ±2.35	-41.52 ±2.59	5.91 ±0.04	-35.60 ±2.55
D25	-49.87 ±2.24	-8.11 ±3.20	22.8 5±2.48	-5.14 ±0.16	-57.98 ±3.50	17.70 ±2.43	-40.28 ±2.34	7.38 ±0.04	-32.89 ±2.34
D26	-48.49 ±2.39	-26.50 ±3.54	43.55 ±4.08	-5.77 ±0.26	-74.99 ±4.59	37.78 ±3.94	-37.21 ±2.90	6.61 ±0.04	-30.60 ±2.90

D27	-51.08 ±2.56	-16.25 ±3.75	37.64 ±3.12	-5.67 ±0.18	-68.34 ±3.63	31.85 ±2.22	-36.48 ±2.22	5.18 ±0.02	-31.30 ±2.22
D45	-53.71 ±2.21	-19.59 ±4.63	38.57 ±4.18	-6.17 ±0.24	-73.31 ±4.92	32.39 ±4.13	-40.91 ±2.72	6.45 ±0.05	-34.45 ±2.72
D46	-60.70 ±2.77	-24.78 ±3.83	47.08 ±3.62	-6.61 ±0.22	-85.48 ±4.21	40.47 ±3.57	-45.01 ±3.57	6.72 ±0.12	-38.29 ±3.04
D47	-58.76 ±2.45	-16.54 ±7.12	36.32 ±6.49	-6.32 ±0.20	-75.30 ±6.89	30.00 ±6.53	-45.30 ±2.91	12.66 ±0.05	-32.63 ±2.91

Table S11: MM-PB/GBSA binding energy terms of the designed compounds in complex with $FLT3_{D835Y}$ in kcal/mol

Complexes	E_{vdW} (±SD)	E_{EL} (±SD)	E_{GB} (±SD)	E_{SURF} (±SD)	E_{gas} (±SD)	E_{solv} (±SD)	E_{TOTAL} (±SD)	$T\Delta S$	$\Delta G_{MM-PB/GBSA}$ (±SD)
D02	-56.65 ±2.46	-25.14 ±3.73	51.55 ±3.19	-6.22 ±0.18	-81.80 ±3.77	45.33 ±3.14	-36.46 ±2.50	4.08 ±0.03	-32.37 ±2.50
D03	-53.41 ±2.52	-16.81 ±3.41	36.50 ±2.26	-5.82 ±0.21	-70.23 ±4.01	30.67 ±2.21	-39.55 ±2.99	8.69 ±0.50	-30.86 ±2.99
D04	-52.26 ±2.68	-15.57 ±3.48	35.21 ±2.54	-5.77 ±0.24	-67.84 ±4.23	29.43 ±2.48	-38.40 ±2.95	8.28 ±0.04	30.11 ±2.95
D15	-61.94 ±2.76	-22.38 ±3.34	42.83 ±2.57	-6.96 ±0.22	-84.32 ±4.07	35.86 ±2.49	-48.45 ±3.13	8.81 ±0.68	-39.64 ±3.20
D16	-53.33 ±3.05	-17.65 ±4.28	41.24 ±3.09	-5.61 ±0.21	-70.98 ±0.21	35.63 ±3.23	-35.35 ±2.45	6.40 ±0.03	-28.95 ±2.45
D17	-52.98 ±2.72	-11.26 ±3.06	30.85 ±2.45	-5.66 ±0.19	-64.25 ±3.68	25.19 ±2.40	-39.05 ±3.04	9.33 ±0.95	-29.73 ±3.19
D18	-53.07 ±2.73	-11.04 ±2.93	30.31 ±2.21	-5.60 ±0.18	-64.12 ±3.45	24.70 ±2.12	-39.41 ±2.86	4.79 ±0.04	-34.62 ±2.86
D25	-54.72 ±2.46	-7.45 ±3.18	25.12 ±2.97	-5.85 ±0.20	-62.17 ±3.48	19.27 ±2.92	-42.90 ±2.72	6.79 ±0.03	-36.11 ±2.72
D26	-56.41 ±2.41	-17.72 ±5.24	37.69 ±3.46	-3.40 ±0.30	-74.14 ±5.78	34.28 ±3.43	39.86 ±3.18	7.05 ±0.40	-32.80 ±3.20
D27	-52.47 ±2.72	-15.84 ±4.29	35.40 ±3.68	-5.64 ±0.26	-68.31 ±4.87	29.76 ±3.66	-38.55 ±3.21	8.28 ±0.04	-30.26 ±3.21
D45	-50.95 ±2.21	-22.99 ±4.32	42.49 ±3.39	-5.70 ±0.21	-73.94 ±4.25	36.78 ±3.38	-73.94 ±4.25	5.99 ±0.04	-31.17 ±2.58
D46	-60.01 ±2.58	-20.34 ±4.09	43.11 ±3.75	-6.45 ±0.24	-80.36 ±4.58	36.65 ±3.68	-43.71 ±3.01	7.39 ±0.02	-36.31 ±3.01
D47	-57.84 ±2.50	-24.85 ±6.37	45.86 ±6.26	-6.49 ±0.23	-82.70 ±6.88	39.37 ±6.22	-43.33 ±2.69	10.21 ±0.05	-33.11 ±2.69

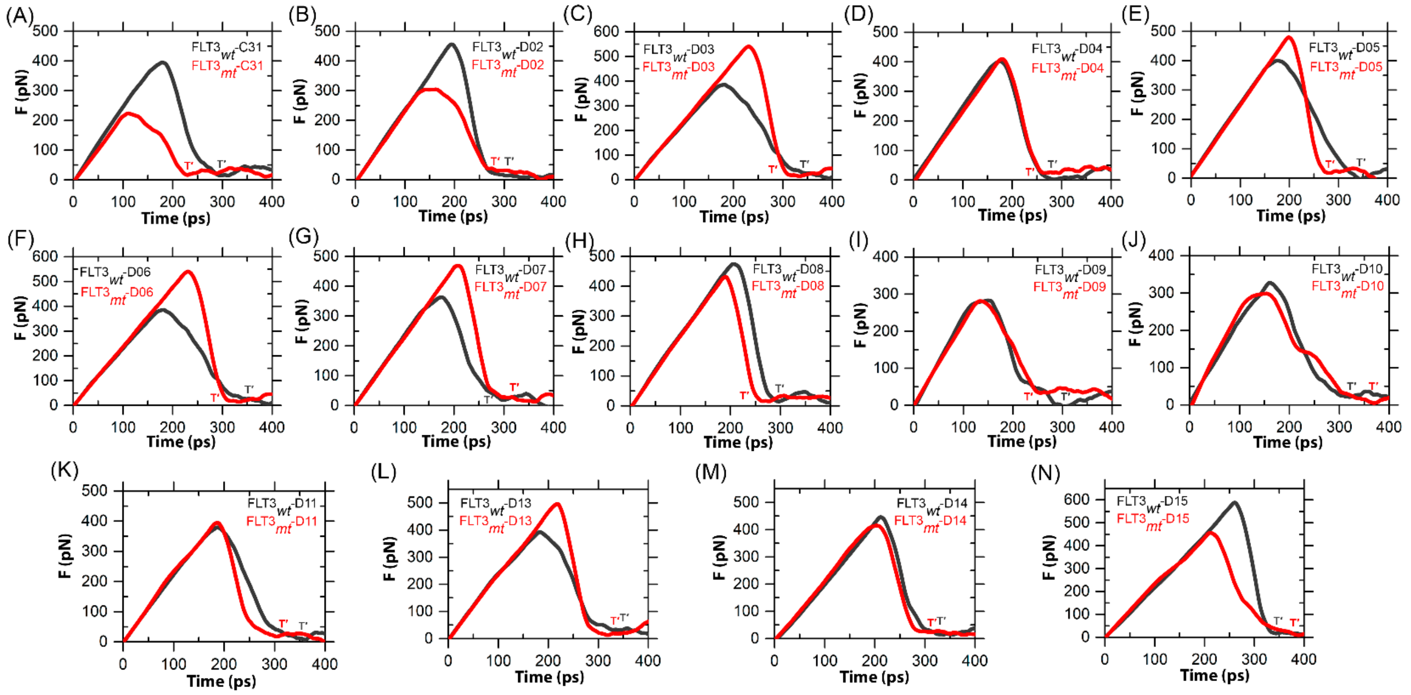

Figure S8. FPL simulation of the designed compounds from the wild type and mutant receptor. The rupture force are shown over the simulation time.

Table S12: LIE binding energy terms of the designed compounds in complex with FLT3 in kcal/mol from the FPL simulation

Compounds	Ligands in Solvent		Ligands in Complex		ΔG_{LIE} (±SD)
	$\frac{1}{2}\Delta E_{coul}$ (±SD)	$\frac{1}{2}\Delta E_{vdW}$ (±SD)	$\frac{1}{2}\Delta E_{coul}$ (±SD)	$\frac{1}{2}\Delta E_{vdW}$ (±SD)	
D02	-26.37 ±1.40	-17.86 ±0.90	-11.47 ±1.40	-5.59 ±0.90	-27.69 ±1.66
D03	-22.71 ±1.70	-19.15 ±1.25	-7.37 ±1.70	-5.29 ±1.25	-29.20 ±2.11
D04	-23.00 ±3.10	-18.47 ±0.65	-6.79 ±3.10	-4.65 ±0.65	-30.02 ±3.16
D15	-23.16 ±1.65	-18.89 ±1.15	-4.63 ±1.65	-4.90 ±1.15	-32.51 ±2.01
D16	-25.50 ±2.75	-18.36 ±1.40	-11.43 ±2.75	-5.69 ±1.40	-26.72 ±3.08
D17	-21.93 ±2.20	-18.15 ±0.80	-8.28 ±2.20	-6.94 ±0.80	-24.86 ±2.34
D18	-21.37 ±1.50	-18.77 ±1.15	-14.02 ±1.50	-6.45 ±1.15	-19.67 ±1.89
D25	-22.85 ±1.40	-18.05 ±0.23	-13.41 ±1.40	-6.09 ±0.23	-21.39 ±1.41
D26	-23.85 ±2.15	-17.77 ±1.30	-6.15 ±2.15	-5.84 ±1.30	-29.63 ±2.70
D27	-24.24 ±1.90	-20.28 ±1.60	-10.08 ±1.90	-6.31 ±1.60	-28.13 ±2.48
D45	-23.77	-19.52	-8.57	-6.31	-28.60

	±1.95	±1.30	±1.95	±1.30	±2.34
D46	-22.21	-21.02	-9.02	-7.57	-26.64
	±4.45	±0.90	±4.45	±0.90	±4.54
D47	-22.21	-22.35	-7.89	-7.68	-28.99
	±2.65	±1.70	±2.65	±1.70	±3.14

Table S13: LIE binding energy terms of the designed compounds in complex with $FLT3_{D835Y}$ in kcal/mol from the FPL simulation

Compounds	Ligands in Solvent		Ligands in Complex		ΔG_{LIE} (±SD)
	$\frac{1}{2}\Delta E_{coul}$ (±SD)	$\frac{1}{2}\Delta E_{vdW}$ (±SD)	$\frac{1}{2}\Delta E_{coul}$ (±SD)	$\frac{1}{2}\Delta E_{vdW}$ (±SD)	
D02	-27.19<br>±3.35	-17.91<br>±0.80	-12.55<br>±1.90	-5.96<br>±1.10	-26.58<br>±3.44
D03	-22.48<br>±2.20	-18.68<br>±1.95	-7.14<br>±2.20	-4.91<br>±1.95	-29.11<br>±2.93
D04	-21.70<br>±2.80	-18.67<br>±1.70	-7.06<br>±2.80	-5.40<br>±1.70	-27.91<br>±3.27
D15	-22.73<br>±2.25	-18.86<br>±1.35	-8.76<br>±2.25	-4.74<br>±1.35	-28.09<br>±2.62
D16	-25.94<br>±2.20	-18.59<br>±1.30	-9.92<br>±2.20	-6.37<br>±1.30	-28.23<br>±2.55
D17	-22.28<br>±1.55	-18.10<br>±1.35	-10.09<br>±1.55	-6.28<br>±0.90	-23.40<br>±2.05
D18	-21.02<br>±2.10	-18.78<br>±0.90	-14.40<br>±2.1	-6.28<br>±0.90	-19.11<br>±2.28
D25	-22.78<br>±2.05	-18.70<br>±0.80	-12.39<br>±2.05	-6.16<br>±1.15	-22.71<br>±2.35
D26	-22.85<br>±2.15	-18.70<br>±0.80	-6.83<br>±2.15	-7.43<br>±0.80	-27.29<br>±2.29
D27	-24.08<br>±3.35	-19.03<br>±1.25	-6.83<br>±2.15	-6.37<br>±0.65	-30.54<br>±3.41
D45	-23.77<br>±1.80	-19.72<br>±0.65	-10.81<br>±1.80	-6.14<br>±1.25	-25.84<br>±2.19
D46	-22.05<br>±1.30	-21.72<br>±0.80	-8.87<br>±1.30	-7.93<br>±0.80	-26.97<br>±1.52
D47	-22.21<br>±1.95	-21.86<br>±0.75	-8.35<br>±7.45	-7.45<br>±0.75	-28.27<br>±2.08

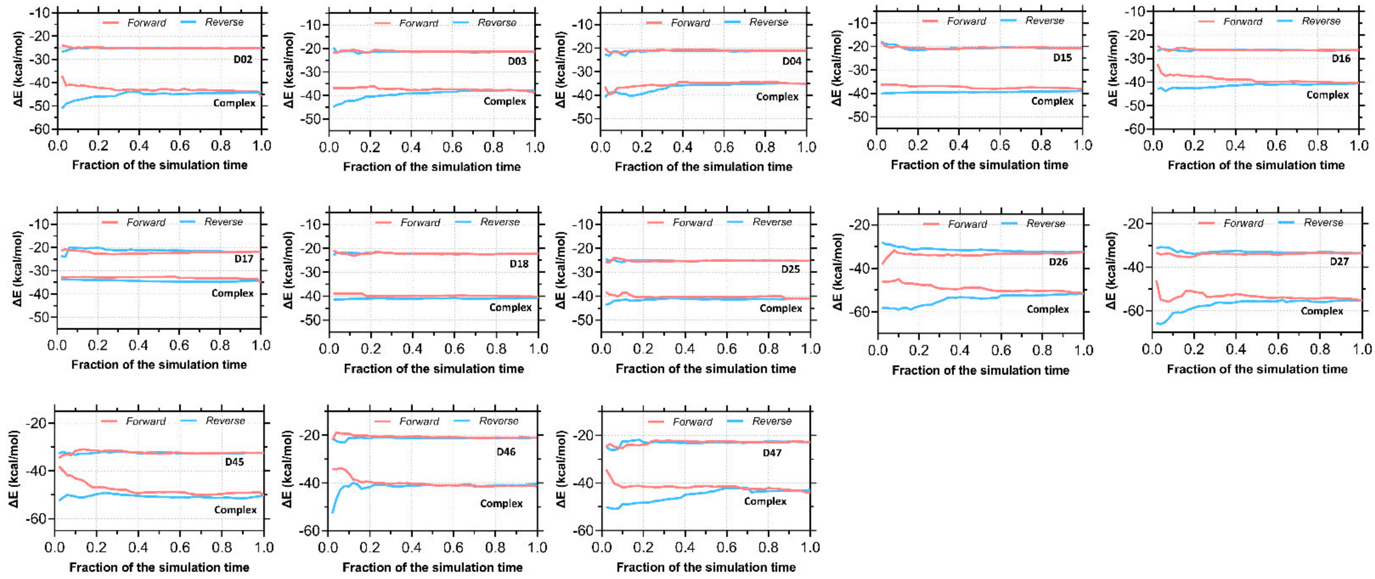

Figure S9. FEP energy convergence plots of the designed compounds targeting wild-type FLT3 over the fraction of simulation time

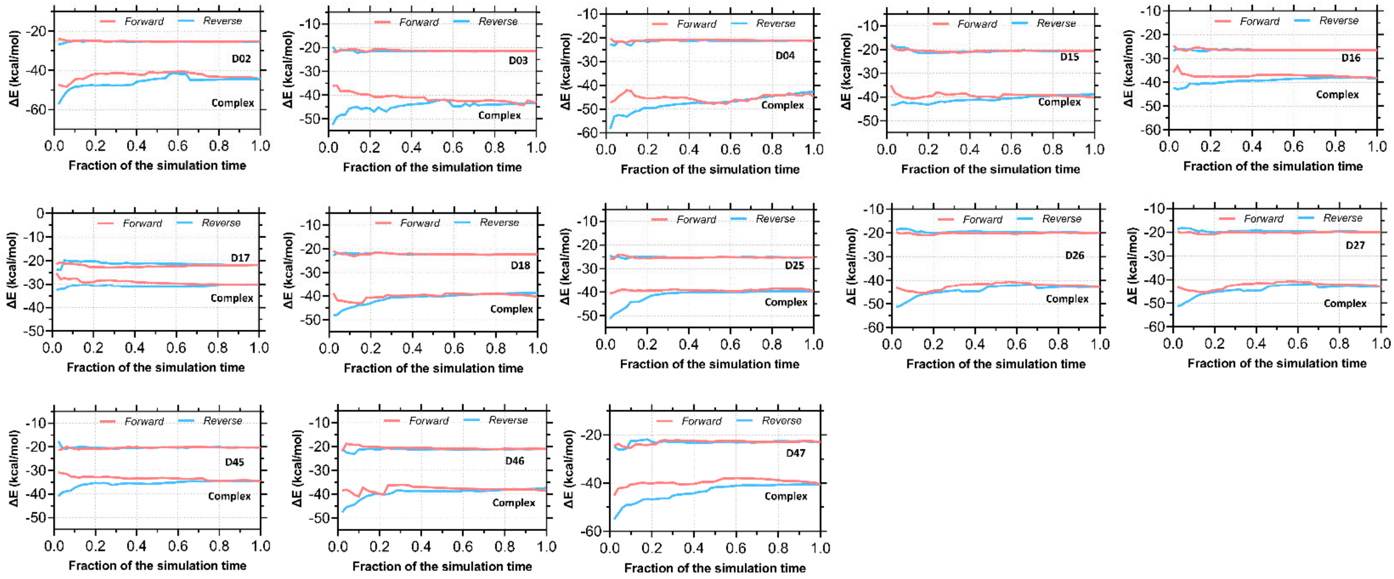

Figure S10. FEP energy convergence plots of the compounds designed to target mutant FLT3 over the fraction of simulation time

Table S14: Calculation of FEP binding energy terms of the designed compounds in the complex with FLT3 variants in kcal/mol over the two-coupling process

Compounds	Ligands in Solvent			FLT3 Ligands in Complex			FLT3	$FLT3_{D835Y}$ Ligands in Complex			$FLT3_{D835Y}$
	ΔE_{coul} (±SD)	ΔE_{vdW} (±SD)	ΔE_{Total} (±SD)	ΔE_{coul} (±SD)	ΔE_{vdW} (±SD)	ΔE_{Total} (±SD)	ΔG_{FEP} (±SD)	ΔE_{coul} (±SD)	ΔE_{vdW} (±SD)	ΔE_{Total} (±SD)	ΔG_{FEP} (±SD)
D02	-23.89 ±0.04	-1.60 ±0.39	-25.49 ±0.99	-23.65 ±0.05	-24.02 ±0.62	-47.68 ±0.63	-22.19 ±1.17	-19.96 ±0.84	-23.28 ±0.09	-43.24 ±0.84	-18.05 ±1.29

D03	-20.26	-1.92	-22.18	-17.84	-18.39	-36.23	-14.05	-17.45	-20.81	-38.27	-16.09
	±0.03	±0.50	±0.50	±0.06	±0.50	±0.50	±0.70	±0.07	±0.26	±0.27	±0.56
D04	-20.44	-0.85	-21.29	-15.68	-19.56	-35.25	-13.96	-18.12	-21.35	-39.47	-18.18
	±0.03	±0.33	±0.33	±0.09	±0.19	±0.21	±0.39	±0.09	±0.45	±0.46	±0.56
D15	-20.52	-0.540	-21.06	-19.24	-19.87	-39.71	-18.65	-18.94	-22.53	-41.47	-20.41
	±0.03	±0.44	±0.44	±0.07	±0.41	±0.41	±0.60	±0.10	±0.25	±0.27	±0.51
D16	-25.66	-1.11	-26.80	-17.45	-18.62	-36.08	-9.28	-16.20	-22.53	-38.73	-11.93
	±0.04	±0.40	±0.40	±0.09	±0.38	±0.38	±0.55	±0.07	±0.83	±0.83	±0.92
D17	-21.93	-0.31	-22.24	-18.77	-16.81	-35.58	-13.34	-13.87	-15.63	-29.51	-7.27
	±0.05	±0.42	±0.43	±0.06	±0.04	±0.64	±0.77	±0.07	±0.49	±0.50	±0.65
D18	-21.63	-1.13	-22.76	-19.13	-21.80	-40.93	-18.17	-19.01	-20.13	-39.14	-16.38
	±0.03	±0.35	±0.35	±0.06	±0.62	±0.63	±0.72	±0.07	±0.26	±0.27	±0.44
D25	-24.18	-1.28	-25.47	-18.78	-21.59	-40.38	-14.91	-19.10	-20.61	-39.71	-14.24
	±0.04	±0.37	±0.37	±0.07	±0.35	±0.36	±0.26	±0.06	±0.28	±0.29	±0.47
D26	-20.02	-0.16	-20.18	-20.84	-11.22	-32.06	-11.88	-17.97	-10.22	-28.20	-8.02
	±0.05	±0.48	±0.49	±0.09	±0.37	±0.38	±0.62	±0.07	±0.66	±0.66	±0.82
D27	-20.26	0.747	-19.51	-9.83	-21.52	-31.35	-11.84	-18.84	-19.18	-38.02	-18.51
	±0.06	±0.51	±0.52	±0.13	±0.57	±0.59	±0.78	±0.12	±0.67	±0.68	±0.85
D45	-20.15	--0.18	-20.33	-15.62	-18.78	-34.40	-14.07	-21.50	-13.29	-34.79	-14.46
	±0.07	±0.51	±0.52	±0.70	±0.26	±0.27	±0.58	±0.21	±0.10	±0.24	±0.57
D46	-20.45	-1.71	-22.16	-19.12	-21.54	-40.67	-18.51	-19.93	-20.51	-44.54	-18.28
	±0.04	±0.50	±0.50	±0.10	±0.44	±0.45	±0.67	±0.29	±0.08	±0.31	±0.58
D47	-20.44	-0.82	-21.27	-20.45	-20.78	-41.23	-19.96	-19.78	-19.77	-39.55	18.28
	±0.04	±0.52	±0.52	±0.06	±0.22	±0.23	±0.56	±0.50	±0.08	±0.50	±0.72
